# First decade anniversary of the United Kingdom National Alkaptonuria Centre

**DOI:** 10.1002/jmd2.12340

**Published:** 2023-01-18

**Authors:** Milad Khedr, Nick Sireau, George Bou‐Gharios, Jim A. Gallagher, Lakshminarayan R. Ranganath

**Affiliations:** ^1^ Department of Clinical Biochemistry and Metabolic Medicine Liverpool University Hospitals NHS Foundation Trusts Liverpool UK; ^2^ AKU Society Cambridge UK; ^3^ William Henry Duncan Building University of Liverpool Liverpool UK

Dear editor,

On April 1st 2012, NHS England Highly Specialised Services (HSS) commissioned the National Alkaptonuria Service for adults in the Department of Clinical Biochemistry & Metabolic Medicine at the Royal Liverpool University Hospital.^1^ The service is called the Robert Gregory National Alkaptonuria Centre (NAC) in memory of the alkaptonuria (AKU) patient who founded the Alkaptonuria Society (AKUS) in 2003.The AKUS is embedded in the NAC and crucial to its functioning and success, ensuring patients are at the centre of the service. The NAC serves as a notable example of power of collaboration between the medical community and patients.

Since 2012, 89 patients have attended the NAC. While patients from England and Scotland can access the service free of charge, patients from Wales, Ireland, Germany, Pakistan, Indonesia and the USA have been treated at the NAC on the basis of a fee. The NAC is a one‐stop service providing assessment, treatment including nitisinone and monitoring of therapy and disease, with visits lasting 3–4 days. NAC provides a full‐range of multidisciplinary specialists (metabolism, rheumatology, cardiology, pain management, dietetic, psychological, ENT, ophthalmology, gait specialists, nuclear medicine, radiology) to manage their condition. An encrypted system PATIENTS KNOW BEST® (PKB) enables real‐time communication between patients and the NAC team.

Nitisinone 2 mg has been used as off‐label therapy in the NAC since 2012, after approval by the HSS based on the published data at the time from the National Institutes of Health, USA.^2^ Experience gained in the NAC has been instrumental in designing and carrying out the DevelopAKUre programme^3^ funded by the European Commission to develop nitisinone as therapy for AKU adults. This consisted of a 4‐week dose‐ranging study of nitisinone in AKU (SONIA 1; suitability of nitisinone in alkaptonuria 1), a cross‐sectional natural history study (SOFIA; subclinical ochronosis features in alkaptonuria) and a 4‐year randomised nitisinone outcomes clinical trial (SONIA 2).^4^, ^5^, ^6^ Successful completion of SONIA 2 resulted in the approval of nitisinone 10 mg as the first disease‐modifying therapy for AKU by the European Medicines Agency in September 2020. The NAC is in the process of switching the nitisinone dose from 2 to 10 mg.^7^ Nitisinone use is associated with inevitable hypertyrosinaemia which has significant side effects such corneal dendritiform keratopathy. Protein restriction is required as soon as nitisinone is started to reduce tyrosine to safe levels. Specialist dietitian input is required to ensure diet is manipulated safely without impacting health (e.g. muscle wasting). There is no evidence of benefit for protein restriction in AKU patients who are not on nitisinone.

The NAC has generated new knowledge about AKU and its management (Table S1); with a few examples being prevalent cataract before and after nitisinone, nitisinone‐associated vitiligo, characterising spine and joint disease and demonstrating lack of cognitive impairment during nitisinone therapy of adults with AKU.^8^, ^9^, ^10^, ^11^ The NAC permitted research collaboration between the NAC and the University of Liverpool, allowing cutting edge research into AKU to be pioneered and carried out with examples including development of conditional mouse models of AKU including liver and kidney knockouts in which new therapies are tested such as tyrosine‐reducing therapies, as well as enzyme replacement therapies involving gene and mRNA administration.^12^ A new mechanism of joint damage in AKU was developed and found to apply in osteoarthritis.^13^

Unachieved goals and further challenges:

The NAC is commissioned to treat patients who 16 or older. We are collaborating with colleagues to study subclinical ochronsis in the paediatric AKU cohort in the UK (called the SOFIA‐Paediatric). It is hoped that this will give an evidence‐based answer as to when to start nitisinone. Home tyrosine monitoring is conveniently done using dried blood spots (DBS) and there is a good agreement between DBS and venous samples in tyrosine measurements. Unfortunately, some of our patients had difficulties in using DBS (dexterity issues in elderly patients due to advanced arthropathy, difficulty in finger pricking due to thick skin, too much/too little blood put on the card). As a result, we have developed an assay system using the Mitra^TM^ sampling device, which offers a more user‐friendly and robust alternative.^14^ Preliminary feedback from patients has been positive and we are in the process of integrating this into the patient pathway.

In the UK, starting as we did in 2003 when there was little in terms of opportunities to identify, support and manage patients with AKU, we have come a long way in redressing the balance, but further work is needed in the years ahead.

## CONFLICT OF INTEREST

Lakshminarayan R. Ranganath received fees for lectures and consultations from Swedish Orphan Biovitrum. Milad Khedr, Nick Sireau, George Bou‐Gharios, and Jim A. Gallagher declare no conflict of interest.

## INFORMED CONSENT

All procedures followed were in accordance with the ethical standards of the responsible committee on human experimentation (institutional and national) and with the Helsinki Declaration of 1975, as revised in 2000 (5). Informed consent was obtained from all patients for being included in the study.

## Supporting information


**TABLE S1.** Contributions to AKU knowledge and patient care over the last decade

## Data Availability

Data sharing is not applicable to this article as no datasets were generated or analysed during the current study.

## References

[1] https://www.england.nhs.uk/wp-content/uploads/2016/11/e06-alkapt-children.pdf

[2] Introne WJ , Perry MB , Troendle J , et al. A 3‐year randomized therapeutic trial of nitisinone in Alkaptonuria. Mol Genet Metab. 2011;103:307‐314. 21620748 10.1016/j.ymgme.2011.04.016 PMC3148330

[3] https://cordis.europa.eu/project/id/304985

[4] Ranganath LR , Milan AM , Hughes AT , et al. Suitability of nitisinone in Alkaptonuria 1 (SONIA 1): an international, multicentre, randomised, open‐label, control controlled, parallel‐group, dose–response study to investigate the effect of once daily nitisinone on 24‐h urinary homogentisic acid excretion in patients with Alkaptonuria after 4 weeks of treatment. Ann Rheum Dis. 2016;75:362‐367. 25475116 10.1136/annrheumdis-2014-206033

[5] Cox TF , Psarelli EE , Taylor S , et al. Subclinical ochronosis features in Alkaptonuria: a cross‐sectional study. BMJ Innov. 2019;5:82‐91.

[6] Ranganath LR , Psarelli EE , Arnoux JB , et al. Suitability of nitisinone in Alkaptonuria 2 (SONIA 2): a randomised study on the efficacy and safety of nitisinone in Alkaptonuria. Lancet Diabetes Endocrinol. 2020;8:762‐772. 32822600 10.1016/S2213-8587(20)30228-X

[7] Ranganath LR , Milan AM , Hughes AT , et al. Comparing nitisinone 2 mg and 10 mg in the treatment of Alkaptonuria: an approach using statistical modelling. JIMD Rep. 2021;63:1‐13. 10.1002/jmd2.12261 PMC8743340 35028273

[8] Ranganath LR , Vinjamuri S , Gallagher JA . Characterising the Alkaptonuria joint and spine phenotype and assessing the effect of homogentisic acid lowering therapy in a large cohort of eighty‐seven patients. J Inherit Metab Dis. 2021;44:666‐676. 33452825 10.1002/jimd.12363

[9] Ranganath LR , Khedr M , Evans LA , Bygott H , Luangrath E , West E . Vitiligo, Alkaptonuria, and nitisinone: a report of four cases and review of the literature. JIMD Rep. 2021;61:1‐9. doi:10.1002/jmd2.12225 PMC8411108 34485014

[10] Ahmad MSZ , Ahmed M , Khedr M , et al. Association of Alkaptonuria and low dose nitisinone therapy with cataract formation in a large cohort of patients. JIMD Rep. 2022;63:351‐360. 35822094 10.1002/jmd2.12288 PMC9259401

[11] Davison AS , Hughes G , Harrold JA , Clarke P , Griffin R , Ranganath L . Long‐term low dose nitisinone therapy in adults with Alkaptonuria shows no cognitive decline or increased severity of depression. JIMD Rep. 2022;63:221‐230. 35433173 10.1002/jmd2.12272 PMC8995840

[12] Hughes JH , Liu K , Plagge A , et al. Conditional targeting in mice reveals that hepatic homogentisate 1,2‐dioxygenase activity is essential in reducing circulating homogentisic acid and for effective therapy in the genetic disease Alkaptonuria. Hum Mol Genet. 2019;28:3928‐3939. 31600782 10.1093/hmg/ddz234 PMC7073386

[13] Gallagher JA , Ranganath LR , Boyde A . Lessons from a rare disease: what does the arthropathy of Alkaptonuria teach us about disease mechanisms in osteoarthritis and ageing of joints? Rheumatology. 2016;55:1151‐1152. 26712212 10.1093/rheumatology/kev401

[14] Taylor JM , Hughes AT , Milan AM , Rudge J , Davison AS , Ranganath LR . Evaluation of the Mitra microsampling device for use with key urinary metabolites in patients with Alkaptonuria. Bioanalysis. 2018;10:1919‐1932. 30412682 10.4155/bio-2018-0193

