# Supplementary material for: First decade anniversary of the United Kingdom National Alkaptonuria Centre
Source: JIMD Rep. 2023 Jan 18;64(2):212–3. doi: 10.1002/jmd2.12340 (PMC9981412; doi:10.1002/jmd2.12340)
Supplement: Supplementary file 1 — TABLE S1. Contributions to AKU knowledge and patient care over the last decade [file JMD2-64-212-s001.docx]

| **Supplementary Table1:** Contributions to AKU knowledge and patient care over the last decade |
| --- |
| Generating and sharing new knowledge to improve understanding of AKU with over 120 publications |
| Planning and successful completion of the DevelopAKUre programme with funding from the European Commission to develop nitisinone as therapy for AKU adults. This culminated in approval of nitisinone 10mg as the first disease-modifying therapy for AKU by the European Medicines Agency in September 2020. |
| Annual Patient workshops organised by the AKU society: to bring together AKU patients and inform them about advances in research and treatment as well as information and interactive sessions to help them better manage their AKU each day. |
| International scientific workshops organised by the AKU society. The latest one was in Brussels in May 2022 that brought together more than 80 scientists and clinicians to discuss recent advances in AKU research and treatment, such as work on a new gene therapy. |
| Development an e-learning module on AKU specifically for nurses. This piece of work was collaboration between NAC, AKUS and the Royal College of Nursing (RCN). The module launched in November 2019 and was awarded RCN accreditation. |
| Cutting edge research in collaboration with the University of Liverpool |
| 'Body and Mind' project set up by the AKU Society to support the mental wellbeing AKU patients through the Covid-19 pandemic. |
| Working with the non-profit group ‘Rareminds’, the AKU Society was able to offer a free remote counselling service to all our patients, family members and AKU community. An eight-week remote mindfulness course was also offered to patients to provide with techniques that could be used to help them manage their ongoing chronic pain and anxiety on a daily basis. |
